# Pain, comorbidities, and clinical decision-making: conceptualization, development, and pilot testing of the Pain in Aging, Educational Assessment of Need instrument

**DOI:** 10.3389/fpain.2024.1254792

**Published:** 2024-02-22

**Authors:** Bernadette C. Siaton, Beth B. Hogans, Laura A. Frey-Law, Lana M. Brown, Christopher M. Herndon, Luis F. Buenaver

**Affiliations:** ^1^ Division of Rheumatology and Clinical Immunology, Department of Medicine, University of Maryland School of Medicine, Baltimore, MD, United States; ^2^ Geriatric Research Education and Clinical Center, VA Maryland Health Care System, Baltimore, MD, United States; ^3^ Department of Neurology, Johns Hopkins School of Medicine, Baltimore, MD, United States; ^4^ Department of Physical Therapy and Rehabilitative Science, University of Iowa Carver College of Medicine, Iowa City, IA, United States; ^5^ Geriatric Research Education and Clinical Center, Central Arkansas Veterans Healthcare System, Little Rock, AR, United States; ^6^ Department of Pharmacy Practice, Southern Illinois University Edwardsville School of Pharmacy, Edwardsville, IL, United States; ^7^ Department of Family and Community Medicine, St. Louis University School of Medicine, St. Louis, MO, United States; ^8^ Department of Psychiatry and Behavioral Sciences, Johns Hopkins School of Medicine, Baltimore, MD, United States

**Keywords:** interprofessional, interdisciplinary, pain education, clinical decision-making, geriatric, multimorbidity, survey instrument, chronic pain

## Abstract

**Introduction:**

Pain is highly prevalent in older adults and often contextualized by multiple clinical conditions (pain comorbidities). Pain comorbidities increase with age and this makes clinical decisions more complex. To address gaps in clinical training and geriatric pain management, we established the Pain in Aging—Educational Assessment of Need (PAEAN) project to appraise the impacts of medical and mental health conditions on clinical decision-making regarding older adults with pain. We here report development and pilot testing of the PAEAN survey instrument to assess clinician perspectives.

**Methods:**

Mixed-methods approaches were used. Scoping review methodology was applied to appraise both research literature and selected Medicare-based data. A geographically and professionally diverse interprofessional advisory panel of experts in pain research, medical education, and geriatrics was formed to advise development of the list of pain comorbidities potentially impacting healthcare professional clinical decision-making. A survey instrument was developed, and pilot tested by diverse licensed healthcare practitioners from 2 institutions. Respondents were asked to rate agreement regarding clinical decision-making impact using a 5-point Likert scale. Items were scored for percent agreement.

**Results:**

Scoping reviews indicated that pain conditions and comorbidities are prevalent in older adults but not universally recognized. We found no research literature directly guiding pain educators in designing pain education modules that mirror older adult clinical complexity. The interprofessional advisory panel identified 26 common clinical conditions for inclusion in the pilot PAEAN instrument. Conditions fell into three main categories: “major medical”, i.e., cardio-vascular-pulmonary; metabolic; and neuropsychiatric/age-related. The instrument was pilot tested by surveying clinically active healthcare providers, e.g., physicians, nurse practitioners, who all responded completely. Median survey completion time was less than 3 min.

**Conclusion:**

This study, developing and pilot testing our “Pain in Aging—Educational Assessment of Need” (PAEAN) instrument, suggests that 1) many clinical conditions impact pain clinical decision-making, and 2) surveying healthcare practitioners about the impact of pain comorbidities on clinical decision-making for older adults is highly feasible. Given the challenges intrinsic to safe and effective clinical care of older adults with pain, and attendant risks, together with the paucity of existing relevant work, much more education and research are needed.

## Introduction

Pain-associated conditions are prevalent in older adults who often experience high rates of medical and mental health conditions, i.e., pain comorbidities (1, 2). A range of professionals provide healthcare services to older adults; current models conceptualize this care in terms of interprofessional collaboration and view this care through the lens of interacting health conditions, i.e., multimorbidity, and systems of care, which taken together comprise multicomplexity (3–9). The multicomplexity intrinsic to healthcare for older adults increases the cognitive challenges which professional practitioners face in clinical decision-making (10–12). This is especially relevant with regards to pain management where failure to acknowledge and address the impacts of comorbidities and multicomplexity in the care of older adults may potentially diminish the effectiveness of educational efforts (13–18). At present, there is no evidence-based framework representing the real-world complexity of older adults living with pain and sufficient to support the construction of pain education modules for healthcare professionals (19).

Pain is so common in older adults that some have proposed that pain is a part of aging (20, 21). Others have argued that pain declines with age; however, the Global Burden of Disease studies indicate that pain rates rise steadily with age to decline only very late in life (2, 22, 23). The most prevalent pain-associated conditions affecting older adults relate to osteoarthritis, but other mechanisms, such as poor sleep quality, comorbid depression, and decreased recruitment of endogenous analgesia may contribute (8, 20, 24–26). Pain in older adults, separate from interactions with other conditions has intrinsic complexity (20, 27). This is compounded by the presence of comorbidities and the extent to which comorbidities increase the challenge of clinical decision-making in managing the pain of older adults is not well understood; the importance of understanding the context of pain has been highlighted by the IASP curricula (28–30). At the level of a single comorbid diagnosis, some diagnoses are known to be both highly prevalent and impactful in choosing therapies for older adults with pain (31, 32). Depression, for example, has a complex relation to pain, potentially increasing risks for and being increased by pain, as well as impacting compliance with pain therapies (25, 33–35). Heart disease, cerebrovascular disease, dementia, renal failure, and hepatic failure can all impact medication safety (36).

We and others have noted the need for intentionally designed educational curricula to address pain in older adults to prepare current and future healthcare practitioners (14, 37, 38). In order to create relevant and effective curricula, it is important to consider the real-world context in which practitioners treat chronic pain, i.e., a patient's medical and/or mental health comorbidities and the pharmacologic therapies used to treat them; in a formal curriculum development framework, this is a foundational preparation step termed “task assessment” (39). Needs assessment of the clinical contexts of pain management in older adults still requires additional refinement (40). Nonetheless, it is likely that comorbidities directly affect clinical decision-making in the treatment of chronic pain (1). In this study, we sought to formulate, and pilot test an instrument designed to assess the extent to which healthcare practitioners perceive common pain comorbidities as impacting decision-making pertaining to the treatment of pain in older adults.

## Methods

This study followed an intentional mixed-methods process incorporating and integrating evidence from (1) an informationist-supported multi-step literature search, (2) review of Medicare-based population-level data about pain and comorbidities in older adults, and (3) advice from an interprofessional, subject matter expert panel (41–43).

### Pain comorbidities literature search

A multi-stage approach was required for the literature search of pain conditions and comorbidities. An initial literature search, directed by a health science librarian, sought to examine the prevalence of chronic pain comorbidities in older adults and used the search terms, “prevalence AND chronic pain AND comorbid or comorbidities.” Our target was to identify relevant literature encompassing pain-associated conditions with high prevalence in older adults, i.e., conditions for which prevalence was estimated to exceed 100 per 100,000. This search yielded 118 results, which were individual reviewed by BH and BS for relevance. A preliminary list of comorbidities was created after review of the articles with highest relevance, Table 1. Reference lists from these articles were reviewed to identify additional articles of interest. The references from these additional articles were reviewed to find further additional relevant articles. Comorbidities from the articles selected in this manner were evaluated. Another literature search sought to examine the prevalence of chronic pain and medical comorbidities in older adults. A health science librarian used the following pain terms (in alphabetical order), “Chronic pain, Chronic widespread pain, Diabetic peripheral neuropathy, Diabetic neuropathies, Fibromyalgia, Headache, headache disorders, Hip pain, Knee pain, Low back pain, Lower back pain, Neck pain, Patellofemoral pain syndrome, Shoulder pain” along with the following comorbidity terms: “Comorbidity terms: Comorbid, Co-morbid, Complexity, Co-diagnosis, Multimorbid, Multi-morbid.” A search utilizing pairs of chronic pain conditions and medical comorbidity terms yielded 104 unique literature results. Two study team members (BH and BS) reviewed results for relevance, and additional comorbidities were added to the preliminary list.

**Table 1:** Scoping literature review—identification of potential key elements.

Preliminary literature review		Secondary literature review	
“Medical” conditions	“Neuropsyciatric conditions”	“Medical” conditions	“Neuropsychiatric conditions”
Obesity (44–46)	Anxiety (46, 47)	CHF (8, 24, 48–50)	Dizziness
Substance use (51)	Depression (44–46)	Stroke (8, 24, 26, 49)	Falls (24)
HTN (51)	Dementia (52)	HTN (8, 24, 26, 32, 48, 50, 53, 54)	Dementia (8, 24, 26)
HLD (51)	Pain conditions	CAD/IHD (8, 24, 26, 32, 48, 49, 54)	Delirium
Lung disease (51)	Headache (55)	Atrial Fibrillation (8, 24, 32)	Depression (8, 24, 49, 50, 54)
Diabetes (51)	Osteoarthritis (55)	HLD (8, 24, 32, 54)	Anxiety (24, 49, 54)
Heart disease (13)	Neck pain (55)	Anemia (24, 50)	OUD (24, 50)
Stomach disease (44)	Low back pain (55)	Asthma (8, 24, 32, 49, 54)	Pain conditions
	Polyneuropathy (55)	COPD (8, 24, 26, 49, 50, 54)	Headache (24)
	Fibromyalgia (51)	OSA (24)	Cervical spine pain (24)
	Chronic pain (44–47, 52, 55)	DM (8, 24, 32, 48–50, 54)	Thoracic spine pain
	Widespread pain (46)	Obesity (32)	Low Back pain (24, 32, 48–50, 54)
	TMD (8)	GERD (24, 32, 54)	Fibromyalgia (53)
		Hypothyroidism (24, 54)	Myalgias
		Renal Impairment (8, 24, 50)	DMPN (26)
		Hepatic Impairment (24)	Shoulder pain
		Osteoporosis (8, 32)	Hip pain (48)
		Vit. B12 Deficiency (24)	Knee pain (48)
		Vit. D Deficiency (24)	

*HTN*, Hypertension; *HLD*, Hyperlipidemia; *TMD*, temporomandibular joint disorder; *CHF*, Congestive Heart Failure; *CAD/IHD*, Coronary Artery Disease/Ischemic Heart Disease; *COPD*, Chronic Obstructive Pulmonary Disease; *OSA*, Obstructive Sleep Apnea; *DM*, Diabetes Mellitus; *GERD*, Gastro-Esophageal Reflux Disease; *Vit.*, Vitamin; *OUD*, Opioid Use Disorder; *DMPN*, Diabetic Peripheral Neuropathy.

### Population-level pain comorbidities data

**Figure 1:**
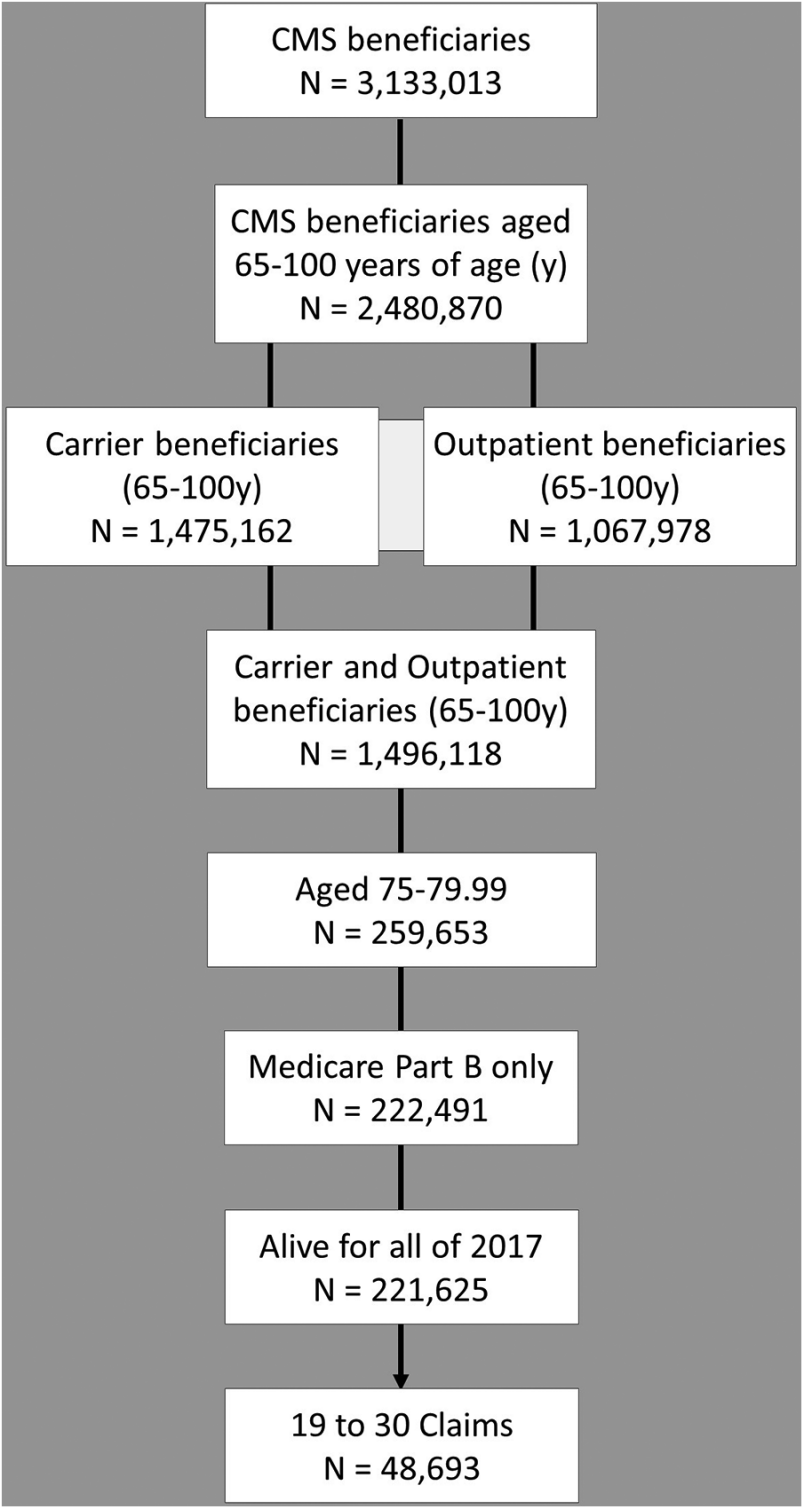
Population flow diagram for scoping data review. Medicare beneficiaries meeting study criteria were selected as illustrated.

The 2017 Center for Medicare and Medicaid Services 5% standard analytical sample of carrier claims data were queried, as previously described, for the 20 most prevalent medical conditions in elderly adults (56). Our previously described data extraction approach was modified as follows, in brief, the extraction followed the sequence illustrated in the population flow chart, Figure 1. The total 2017 CMS beneficiaries numbered approximately 3 million, these were initially limited to those aged 65–100 who numbered approximately 2.5 million. The beneficiaries with claims present in either the Carrier or the Outpatient files were included for a total of approximately 1.5 million. This was further limited to the population of those 75–80 years old, participating in Medicare Part B but not in Part C, and alive for all 12 months of 2017, and the population of those with claims near the median, i.e., 40th–60th percentile for claims (56–60). Age was limited as we observe marked increases in variation in Medicare program usage and mortality at the younger and older extremes of old age respectively (57). The age cohort selected for study does span the median age for U.S. older adults (over 65 years old). Claims were limited as we have observed that beneficiaries with lower claims per year have lower diagnostic rates for common conditions, and those with many claims per year may have higher rates. The claims cohort spanning the median was selected as we seek here to define the properties of a “median” older adult population (57). The final study population for this unadjusted appraisal of rates of common pain conditions and common pain comorbidities was just under 50,000.

### Interprofessional advisory panel

Through directed invitation, we assembled a geographically and professionally diverse subject matter expert interprofessional advisory panel (IAP) consisting of eight nationally recognized experts in pain care, health professions education, and gerontology. Criteria for invitation included: established expertise in a relevant area: academic appointment, presentation at national meetings, and peer-reviewed publications; interest in interprofessional collaboration, and responsiveness. Eight professionals were invited initially; all except one accepted the invitation who provided a reference to another, like professional who accepted our invitation. All professionals remained in contact throughout the study development period except for one physician who stepped back midway in the context of a job change. The IAP included 4 physicians (internist, neurologist, psychiatrist, and rheumatologist), one registered nurse, one pharmacist, one clinical psychologist, and one physical therapist, Table 2. The group met virtually to discuss the potential questions of interest, evaluate and comment on extracts of clinical data and review results of the literature search. The goals of the IAP were to develop a list of “high value” pain comorbidities based on prevalence and potential impact on care, and to advise on survey instrument construction.

**Table 2:** PAEAN interprofessional advisory panel (IAP).

Clinical and research training	Current role	Region	Expertise
Internal medicine, Rheumatology, Medical education	Associate Professor, Clinician educator, Fellowship director^a^	Mid-Atlantic	Rheumatology, medical education, program building, clinical decision-making
Neurology, Clinical neurophysiology, pain, physiology, biomathematics	Associate Director for Education (Geriatrics), Associate Professor, Principal investigator^b^	Mid-Atlantic	Neuropathy, low back pain, pain education, geriatric data science, interprofessionalism
Internal medicine, Medical education	Dean, Professor^c^	Midwest	Medical education
Pharmacy, Education, Research	Professor, Principal investigator^d^	Midwest	Pain education, pharmacology
Physical therapy, Research	Professor, Principal investigator^e^	Midwest	Pain and physical activity, rehabilitation science, aging
Psychiatry, Geriatric psychiatry, Research	Professor, Geriatric psychiatrist, Principal Investigator^f^	Pacific Northwest	Geriatric psychiatry, mental health
Clinical psychiatry, Sleep medicine, Clinical research	Associate professor, Principal investigator^g^	Mid-Atlantic	Clinical psychology, sleep, pain
Nursing and Geriatrics, Behavior change research	Associate Director for Education and Evaluation (Geriatrics)^h^	South Central	Substance use and behavior change

Details of current roles:

^a^ Attending physician and clinical preceptor, VAMHCS and University of Maryland Medical System; Program Director, Rheumatology Fellowship; Associate Program Director, Internal Medicine Residency; project co-PI.

^b^ Associate Professor, Neurology, Johns Hopkins School of Medicine; Associate Director, Geriatric Research, Education, and Clinical Center, VA Maryland; Lead Site Investigator, SCEPTER study; Program director, Office of Research and Development Program Summer Research Program, VAMHCS; Attending clinician and clinical preceptor, VA Neurology inpatient consultation and outpatient clinic; project co-PI.

^c^ Professor, Department of Medicine, Professor of Geriatrics, Department of Family Medicine and Community Health, Vice Dean of Medical Education, Case Western Reserve University School of Medicine.

^d^ Professor, School of Pharmacy at Southern Illinois University Edwardsville, Associate Professor, Department of Family and Community Medicine, St. Louis University School of Medicine.

^e^ Professor, Department of Physical Therapy and Rehabilitative Science, Physical Therapist, University of Iowa Carver College of Medicine.

^f^ Professor, Department of Psychiatry and Behavioral Sciences, University of Washington.

^g^ Associate Professor, clinical psychologist, Director, Behavioral Sleep Medicine Program, Department of Psychiatry and Behavioral Sciences, Johns Hopkins School of Medicine.

^h^ Associate Director/Education and Evaluation, Geriatric Research, Education, and Clinical Center, Central Arkansas Veterans Healthcare System.

### Integration of literature review and data extracts with input from advisory panel

These results were combined with a list of common medical conditions found through literature searches above. Using an iterative review process, a final list of 26 medical conditions and 13 chronic pain conditions were included in the final survey. The instrument prompt was presented to the interprofessional advisory panel and revised for clarity and concision.

### Survey instrument pilot testing

An interprofessional and multi-disciplinary group of 8 board-certified healthcare practitioners, including clinically active physicians and nurse practitioners providing general medical or geriatrics care, from 2 affiliated institutions (University of Maryland Medical Center and the VA Maryland Health Care System) were invited to pilot the survey. No members of the IAP were included in this group. Respondents were asked to rate their agreement with: “This is a common condition in older adults and potentially impacts my decision-making regarding treatment of pain” using a 5-point Likert scale. Individual conditions were scored in terms of the percentage of respondents who agreed or strongly agreed with the prompt statement. Respondents were asked to provide demographic information on their specialty, practice setting, professional title, institution, and number of years in practice.

We scored the survey results as the percent of respondents selecting “agree” or “strongly” agree. Data were processed using Excel (Microsoft) and SAS (Cary, NC). Results are reported as average percent agreement. This pilot study was not powered to detect differences between conditions but was intended to test the instrument for feasibility of use.

This study was approved by the University of Maryland Medical Center IRB and the VA Maryland Health Care System Research and Development Committee.

## Results

### Literature review

Extensive literature review did not identify articles directly addressing the impact of common comorbidities on pain treatment decision-making in older adults. A small number of articles addressed the occurrence of medical and mental health comorbidities in those with pain-associated diagnoses or pain states (reporting chronic or acute pain), Table 3. Study methodologies were largely cross sectional, with information gathering through population-based survey or health system database analysis or both.

**Table 3:** Literature results summary.

Authors and year of publication	Methodology and ascertainment	Population	Pain conditions or states of interest	Comorbidities included	Number of study subjects	Study sponsorship
Ohayon MM, Stingl JC (61)	Prospective phone survey of a random, representative sample of the German national population.	National population, ≥15 years old	Prevalence of acute or chronic pain; neuropathic and non-neuropathic features	Psychiatric and medical comorbidities	3,011 (701 ≥ 65 years old (older adult data not reported separately)	Pfizer
Häuser W, et al. (44)	Prospective home visit survey of a randomly selected sample, designed to represent the German population in terms of age, sex, and education	National population including those willing to respond completely, ≥15 years old	Chronic Pain and Persistent Bodily Pain	“generic self-administered comorbidity questionnaire (SCQ)… to assess common diseases that might impact functioning (62). The SCQ asks about the presence, treatment, and functional limitations of 12 common diseases and 3 additional nonspecified medical problems.”	2,850 [of 4,508 attempted (Mean age 49.7 years, older adult data not reported separately)]	Multiple sources including federal, academic and NGO sources. Two authors with multiple pharmaceutical funding disclosures
Ramanathan S, et al. (32)	Prospective study of a randomly selected sample, designed to represent the Australian population. Phone recruitment followed by phone surveys and consent to medical records review	National population including those willing to consent to phone surveys and medical records review, ≥15 years old.	Low back pain	22 “CareTrack” study comorbidities, through medical records review (primary outcome) and patient phone survey (secondary outcome) (63). Reported rates of 10 most common of these.	164 (medical records review) and 799 (phone survey) (of 15,292 initially contacted) [107 ≥ 55 years old (older adult data not reported separately)]	Federal sources
Lamerato LE, et al. (45)	Retrospective, observational study of electronic health records (EHR) records and claims in a large healthcare system in Detroit	Patients of a regional health care system “…identified based on the presence of ≥2 ICD-9-CM codes at least 30 days apart for the conditions of interest….”	“…derived from a [prior] study… supplemented by conditions addi-tionally identified… for a total of 24 chronic pain conditions.”	Charlson comorbidity conditions (64). A companion paper examined healthcare utilization (costs) and number of Charlson comorbidities using unadjusted quartile analysis (65).	127,317 [4,950 ≥ 65 years old (older adult data not reported separately)]	Pfizer
Price-Haywood EG, et al. (66)	Prospective observation of convenience sample: patients of “…primary care providers (internal medicine or family medicine) practicing in 36 clinic locations across southeast Louisiana in an integrated delivery system” prescribed opioids for chronic noncancer pain during ascertainment period.	Patients of a regional health care system “…age 18 years and older… received opioid prescriptions for 3 of the previous 4 months (chronic opioid therapy), and no active diagnosis of cancer …”	Chronic noncancer pain: “…most common pain syndromes included neck/back/knee pain, arthritis, and fibromyalgia…”	Charlson comorbidity index and comorbid mental health conditions, e.g., depression, anxiety, substance use disorder	14,221	Federal sources
Higgins DM, et al. (46)	Secondary analysis of a prospective convenience sample derived from a U.S. federal health system: “…national sample consisted of 45,477 overweight/ obese Veterans who received healthcare in VHA facilities and expressed interest in weight management.”	Patients of a federal health system for military Veterans with “self-reported weight and height consistent with a BMI ≥25 and completed the MOVE!23 survey in 2006.”	Obesity/overweight was the exposure, rate of pain-associated conditions and rates of multimorbidity were the outcomes	“…20 possible responses for medical comorbidities … and 13 possible responses for mental health comorbidities….” (67).	32,743 reporting ≥1 pain-associated condition of 45,477 ascertained through the Move!23 survey	None listed, likely received salary support from the Veterans Health Administration

*NGO*, Non-Governmental Organization; *ICD-9-CM*, International Classification of Diseases, 9th revision, clinical modification; *U.S.*, United States; *VHA*, (U.S.) Veterans Health Administration; *BMI*, body mass index; MOVE!23, a survey specific to a VHA health program.

Disclosures:

Ohayon et al: Funding for this study was provided by Pfizer Inc. The supporting entity had no role in the design and conduct of the study (collection, management, analysis) nor in the interpretation of the data. The supporting entity has not seen the manuscript and had no role in the decision to submit the paper for publication. The author had full access to all of the data in the study and takes responsibility for the integrity of the data and the accuracy of the data analysis.

Lamerato et al: Disclosures: LEL and RD received research funding from Pfizer and are employees of Henry Ford Health System. JM, PP, GZ, and RHD are full-time employees of Pfizer. Acknowledgements: The study was sponsored by Pfizer Inc. Editorial support was provided by E. Jay Bienen and was funded by Pfizer Inc.

Hauser et al: Supported by internal funds of the Department of Medical Psychology and Medical Sociology of the University of Leipzig and the Department of Psychosomatic Medicine and Psychotherapy of the Technische Universität München, by funds from the German Association of Interdisciplinary Pain Therapy, and by grant 01EO1001 from the German Federal Ministry of Education and Research. W.H. received a consulting honorarium by Daiichi Sankyo and honoraria for educational lectures by Abbott, MSD Sharp & Dhome, and Pfizer within the previous 3 years. P.H. received honoraria for educational lectures by Eli Lilly and Novartis within the previous 3 years. The remaining authors declare no conflict of interest.

Ramanathan et al: The Australian National Health and Medical Research Council (Program Grant No 568612) funds were received in support of this work through a national competitive grant application process. The funder played no role in the design, execution or dissemination of this research.

Price-Haywood et al: This study was supported by 1R01DA045029-01 from the National Institute on Drug Abuse of the National Institutes of Health (NIH). The ideas expressed in this article are the sole responsibility of the authors and do not necessarily represent the official views of NIH.

Higgins et al: The authors have declared that no competing interests exist. [It is noted that several of the authors are employed by the Veterans Health Administration.]

#### Population-based survey studies

Ohayon and colleagues, using population-based phone survey methods, evaluated comorbidities in relation to acute vs. chronic and neuropathic vs. non-neuropathic pain (61). Survey respondents reporting obesity, diabetes, hypertension, and diseases of the cerebrovascular system, nervous system or blood had increased risk for neuropathic pain (61). Those who reported depression were 3-fold more likely to have non-neuropathic pain and 6-fold more likely to have neuropathic pain compared to those without depression. Häuser and colleagues, using population-based home visit methods, evaluated comorbidities in relation to cancer-related vs. non-cancer chronic pain and chronic disabling vs. chronic non-disabling pain (44). The investigators reported that depression was highly associated with chronic pain, as were stomach disease, rheumatic disease, obesity, and heart disease. Ramanathan and colleagues conducted a population-based ascertainment of participants consenting to survey and medical record review of persons reporting low back pain (32). The investigators observed that persons with low back pain had more medical comorbidities and those with more comorbidities described poorer health status. The presence of pain comorbidities increased the risk for provider non-compliance with 9 out of 10 quality indicators, including documentation of a medical history, performance of a physical or neurological examination, and assessment for infection or cancer (32).

#### Health records-based data analytics studies

Lamerato and colleagues extracted records for patients of a U.S.-based healthcare delivery system based on diagnosis with at least one of 24 chronic pain-associated conditions (45). Diabetes, chronic pulmonary disease, malignancy, and renal disease were the most prevalent comorbidities in those with chronic pain-associated diagnoses. In a companion paper, the authors present an unadjusted analysis suggesting that those with the highest healthcare costs have higher rates of comorbidities (65). Price-Haywood and colleagues extracted records for patients receiving primary care from a U.S.-based healthcare delivery system based on receipt of opioid prescriptions (66). A high Charlson comorbidity index was associated with a small decrease in the likelihood of providers prescribing opioids while substance use disorder diagnosis was associated with markedly increased likelihood of providers prescribing opioids (66). Higgins and colleagues extracted records for patients in a federal (nation-wide) U.S.-based healthcare delivery system based on participation in a national survey of U.S. veterans undergoing activity modification for weight management (46). The presence of multiple comorbid conditions increased the risks of low back pain and/or arthritis/joint pain with the likelihood of pain diagnoses increasing as the number of comorbid conditions increased, e.g., those veterans with 5 or more comorbid conditions had 7-fold likelihood of having both low back pain and arthritis/joint pain vs. having “no pain” when compared to those in the study with no comorbid conditions. The authors noted that pain comorbidities are likely to increase treatment complexity (46).

### Clinical claims data scoping review

**Figure 2:**
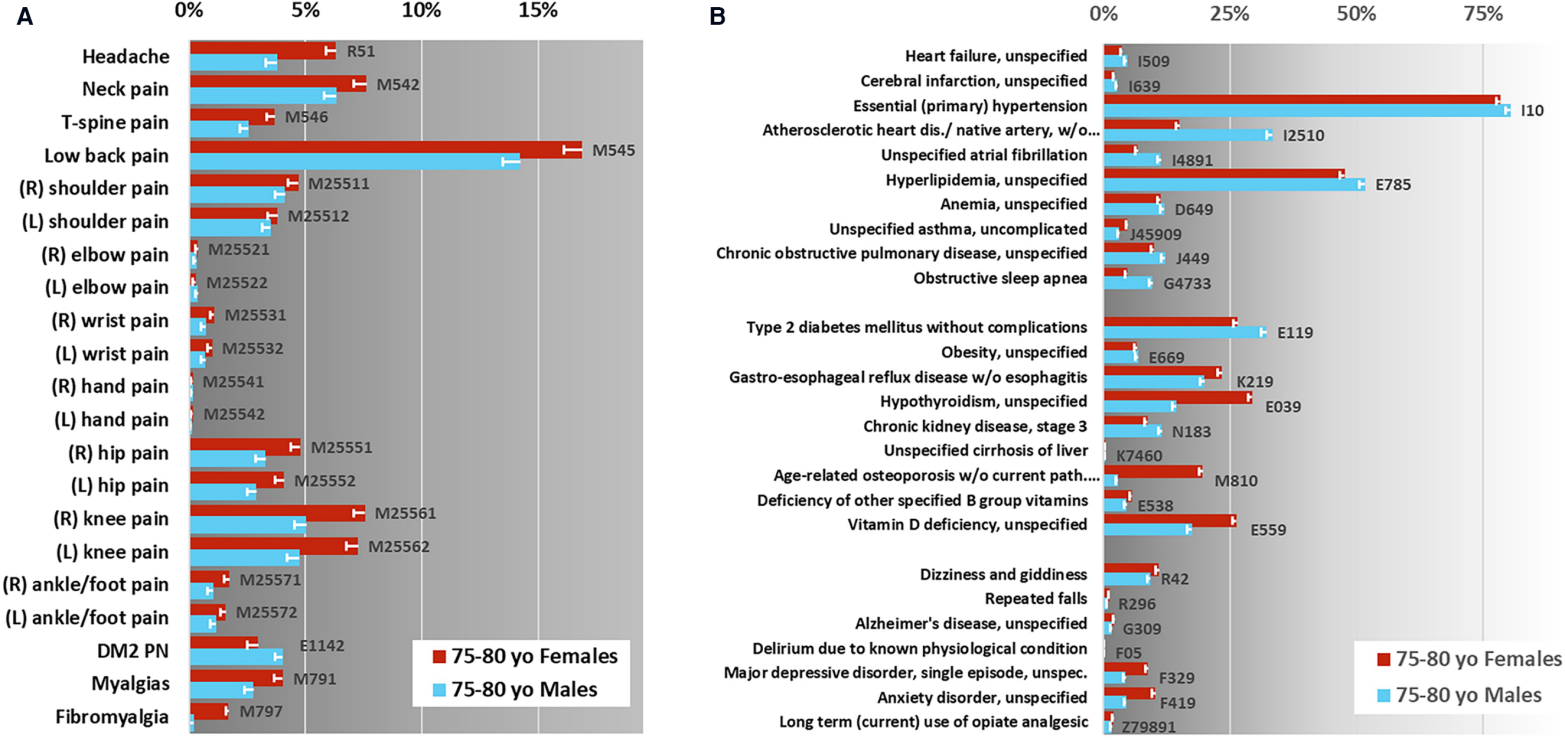
Claims data scoping review—identification of potential key elements. Diagnostic rate estimates for common condition ICD-10 codes in CMS Older adults 75–80 years old (yo). (**A**) Pain condition rate estimates, shown here are the most commonly utilized ICD-10 codes associated with these diagnoses. Pain-associated conditions marked with “*” were included in the assessment instrument. (**B**) Non-pain conditions selected for this study, shown here are the rate estimates for the most commonly used ICD-10 codes associated with each. Error bars indicate corrected 95% confidence interval, *n* = 100, *net p* = 0.05. The gray-scale background in each figure is to alert the viewer to the different x-axis scales.

The data extraction for the purposes of this study included 48,693 Medicare beneficiaries 75–80 years old during 2017 meeting criteria for inclusion, 27,893 (57.3%) were recorded as female gender and 20,798 (42.7%) as male, Figure 1. The average age was 77.38 for females and 77.34 for males. The race and ethnicity distribution, utilizing the Research Triangle Index (%) was Undefined 0.18 and 0.12; White 83.46 and 85.83; Black 7.01 and 5.45; Other 0.81 and 1.15; Hispanic 3.05 and 2.87; Asian American/Pacific Islander 5.05 and 4.18; Native American 0.45 and 0.40 for females and males respectively (68). The rates of common pain diagnoses are shown in Figure 2A. Elbow, wrist, hand, and ankle/foot pain are included to illustrate the relative rates of pain at anatomical sites but these were not included in the group of common conditions which comprised headache, neck pain, thoracic spine pain, low back pain, shoulder, hip, and knee pain; type 2 diabetic polyneuropathy, and fibromyalgia and myalgias (muscle pains). The most common pain code used was M54.5, indicating low back pain. The rates of common medical and mental health diagnoses (comorbidities) selected for study are shown in Figure 2B for females and males. The rates represent the rates of diagnosis based the most common code utilized for each specified condition and are not expected to equate to more systematic appraisals of prevalence, but rather represent a scoping appraisal of ICD-10 code utilization to represent common conditions associated with aging, in the population studied. The most common cardio-vascular-pulmonary condition code used was I10, for Hypertension, which was utilized for over 75% of the studied beneficiaries; the most common metabolic condition code use was E11.9, Type 2 diabetes mellitus, unspecified in males, and E03.9, hypothyroidism, unspecified in females, although hyperlipidemia, unspecified (grouped with cardio-vascular-pulmonary conditions) exceeded both E11.9 and E03.9; and the most commonly used neuropsychiatric/aging-related code was R42, indicating dizziness. The least commonly noted condition incorporated here was hepatic impairment, included due to having a major impact on pain treatment choices, i.e., strict avoidance of acetaminophen and other selected analgesic agents. The extracted data showed some conditions having indications of increased rates in the older adults diagnosed with one or more common pain conditions, e.g., depression, however this was not the focus of this study and further analysis was not pursued.

### Interprofessional advisory panel

The interprofessional advisory panel met 6 times over two years to review and discuss the data obtained and to strategize for and advise the construction of the Pain in Aging, Educational Assessment of Need (PAEAN) survey instrument, Table 2. The inclusion of diverse professional and geographic perspectives increased the number of conditions viewed as comorbid with and potentially significant for pain clinical decision-making in older adults.

### Survey instrument construction

The interprofessional advisory panel (IAP) reviewed and revised the list of conditions integrating literature review and clinical claims data scoping analysis, Table 3. Using a focus group process, respondents iteratively responded with potential comorbidity additions, omissions, and nomenclature until the list finalized. The final decision was to include 26 common clinical [19 medical and 7 neuropsychiatric (mental health)] conditions and 13 common pain-associated conditions in the pilot instrument, Tables 4, 5.

**Table 4:** Initial and final pain-associated condition key element lists.

Initial pain list	Final pain list
Headache	Headache
Cervical spine pain	Cervical spine pain
Thoracic spine pain	Thoracic spine pain
Low back pain	Low Back pain
Fibromyalgia	Fibromyalgia
Myalgias	Myalgias
Diabetic peripheral neuropathy	Diabetic peripheral neuropathy
Focal joint pains	Shoulder pain (right and/or left)
** **	Hip pain (right and/or left)
** **	Knee pain (right and/or left)

**Table 5:** Initial, interim, and final common pain co-morbidity key element lists.

1st comorbidity list	Interim comorbidity list		Final comorbidity list		
HTN	HTN	HLD	Card/vasc/pulm	Metabolic	Neuropsych.
HLD	CAD	CHF	CHF	DM	*Dizziness*
DM	Atrial Fib.	COPD	Stroke	Obesity	*Falls*
Obesity	Asthma	OSA	HTN	GERD	*Dementia*
Depression	Anemia	Obesity	CAD/IHD	Hypothyroid.	*Delirium*
COPD	DM	GERD	Atrial Fib.	Renal Impair.	Depression
Anemia	Renal Failure	Hepatic Failure	HLD	*Hepatic Impair.*	Anxiety
CHF	Osteoporosis	** **	Anemia	Osteoporosis	OUD
OSA	Depression	Anxiety	Asthma	*Vit. B12 Defic.*	** **
Renal Failure	Delirium	Dementia	COPD	*Vit. D Deficiency*	** **
Hepatic Failure	Mild Cognitive Impairment		OSA	** **	** **

Italic font indicates conditions included on the basis on clinical impact based on IAP input. Abbreviations as in Table 1.

**Figure 3:**
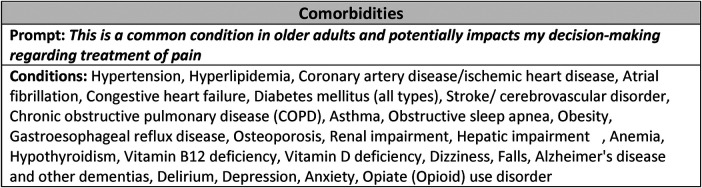
Pilot PAEAN instrument. Shown is the stem (Prompt) and list of conditions included in the final instrument. The instrument is constructed by placing the stem at the top of the page with the list of conditions along the left margin, each with a Likert scale to the right (one scale associated with each condition). The instruction for the instrument was: “Please rate your agreement with the following statement regarding each of the conditions below:” A 5-point Likert scale (strongly disagree to strongly agree) was used.

The draft survey instrument was presented to the IAP for final input and advice. The final version of the instrument consisted of a section for rating pain comorbidities, a section for rating prevalence of common pain conditions, and a section on respondent demographics, Figure 3. Questions about respondent demographics (not reported here) were placed at the end of the instrument in order to improve responder engagement. Respondents reported professional title, institution, years in practice, and primary specialty to validate inclusion.

### Pilot testing

**Figure 4:**
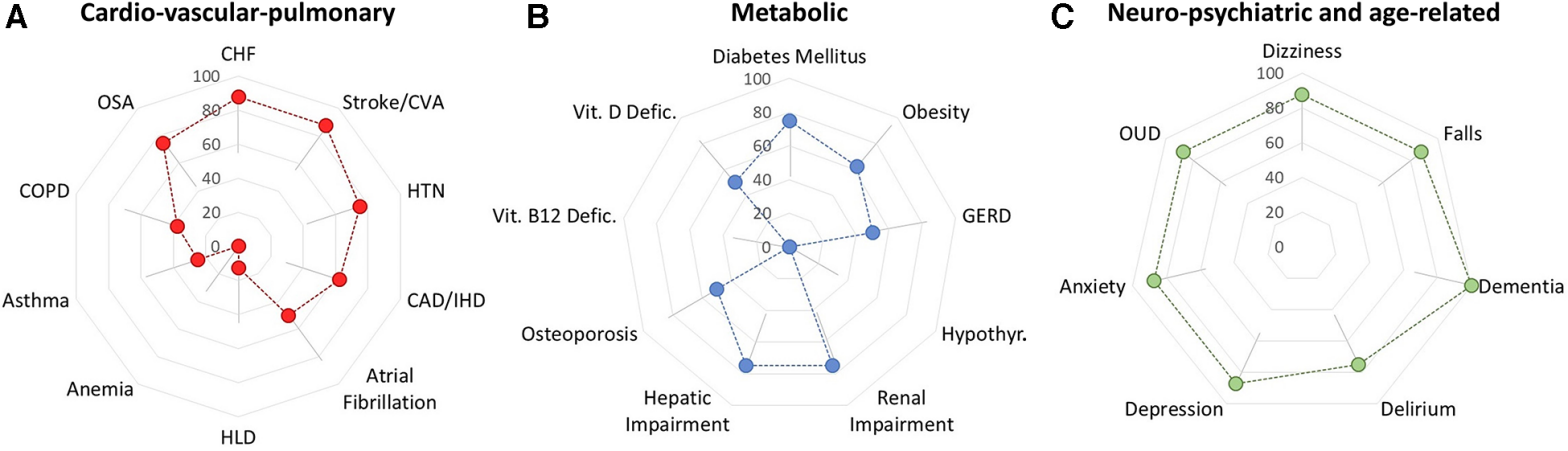
Preliminary assessment of respondent agreement that specified conditions impact pain-related clinical decision-making. (**A**) Cardio-vascular-pulmonary conditions may be viewed a variably impactful. (**B**) Metabolic conditions may be viewed as relatively less impactful although pilot data suggest that diabetes, renal impairment and hepatic impairment may have a strong impact on decision-making. (**C**) Pilot data suggest that neuro-psychiatric conditions have a major impact on pain-related clinical decision-making. Error bars represent the 95% confidence interval, corrected for multiple comparisons, *n* = 26, *net p* = 0.05. For clarity, error bars are shown in one direction only but pertain bidirectionally, with adjustments for floor (near zero) and ceiling (near 100%) effects.

Eight clinically active healthcare practitioners were invited to participate in the pilot survey, all responded to the survey (100% response rate). The median time to complete the survey was 2 min and 45 s, with a range of 1 min and 28 s to 8 min and 34 s. All conditions received a rating from each participant (no missing data). Participants were more likely to select strongly agree than strongly disagree; three conditions had 4 of 8 participants selecting strongly agree, these were “Falls”, “Delirium”, and “Opioid Use Disorder” as impacting pain clinical-decision making. For visualization of the pilot survey results, conditions were grouped together according to main clinical categories as: (1) “Major medical”, i.e., cardio-vascular-pulmonary; (2) “Metabolic”, i.e., involving metabolism, vitamin deficiency syndromes, and endocrine-mediated conditions; and (3) “Neuropsychiatric/age-related”, e.g., falls, dementia. All neuropsychiatric/age-related conditions including dementia and opioid use; selected cardio-vascular-pulmonary conditions, e.g., hypertension and stroke; and selected metabolic conditions, e.g., renal impairment and diabetes mellitus, were rated as impactful (“Agree” or “Strongly Agree”) by most of the practitioners completing the survey, Figure 4.

## Discussion

In this study, we demonstrate that there are many clinical conditions that potentially impact pain clinical decision-making by health care providers caring for older adults, and that this area requires additional study. The outcomes of this study are the pilot instrument as well as a demonstration of the comorbidity data for the study population, the literature review, and an appraisal of the instrument feasibility. The pilot instrument may be used by others, however, in current work we are using a revised stem version, replacing the “and” with “that”. The comorbidity data may be used by others to design pain education cases which incorporate the common and relevant comorbidities of pain in older adults aged 75–80 years. The literature review demonstrated that few articles address the importance of older adult pain comorbidities in clinical decision-making, this was the primary impetus for our study. Finally, we included a demonstration of the type of data that could be obtained with this instrument. We note that this data is pilot data so that the error bars are wide and we do not draw summative conclusions from these values. The time to complete the survey was less than three minutes including demographics items and questions about overall pain condition prevalence. Taken together, we conclude that future studies using this PAEAN instrument are highly feasible and the knowledge gained will improve educational pain case development and ultimately strengthen pain clinical decision-making by those treating older adults. We postulate that medical and mental health comorbidities increase the cognitive burden of pain clinical decision-making, increasing the risk of harms and narrowing the scope of acceptable and feasible therapeutic options (69). The net impact of this cognitive burden remains unknown, but formal needs assessment is essential to the creation of more realistic and clinically useful pain education scenarios (39).

Improved preparation of healthcare providers is a high priority educational goal as the number of older adults is expected to increase (4, 70, 71). In addition to reporting on the conceptualization, development, and pilot testing of a pain clinical decision-making survey instrument, the data presented here are designed to increase awareness of and provide scoping-level data regarding those conditions most likely to increase the complexity of managing persistent pain in older adults (56, 70). Curriculum developers can use information gleaned in this study, together with other research findings, to take pragmatic steps towards improvements in evidence-based pain education initiatives (28, 39, 72–74). As a long-term goal, this study envisions better understanding of and preparation for providers facing real-world challenges in managing pain in and with older adults.

Although it might be assumed that pain clinical decision-making for those treating older adults focuses primarily on pharmacological management, it is important to note that non-pharmacological therapies may result in substantive reductions in pain intensity and interference, although the data specifically, focusing on older adults is limited (23, 75–77). The benefits of nonpharmacological therapies, e.g., exercise, mindfulness-based stress reduction, yoga, and tai chi, may extend to other health benefits, such as improved mobility and balance, reduction of blood pressure, preservation of muscle mass, especially impactful for older adults (78–82). Because of the high prevalence of medical and mental health comorbidities in older adults with pain, a comprehensive approach to pain management, proactively incorporating nonpharmacological as well as pharmacologically based therapies, where appropriate, is often needed and comprehensive approaches should be widely incorporated into pain curricula (69, 83–86).

This study lays the groundwork for considering multi-morbidity in the treatment of chronic pain through an educational curricular development lens. We envision creating a clearer appraisal of the complexities of clinical practice by surveying healthcare professionals who regularly treat older adults many of whom experience persistent pain. These results will help to inform the development of clinical cases, accurately representing patients by accounting for real-world comorbidity and ultimately improving clinical skillfulness at entry to practice and beyond. Educational curricula which ignore the effect of comorbidities and multicomplexity cannot be expected to adequately prepare practitioners for real-world clinical challenges (4, 16, 28, 74, 87).

### Comparison to existing literature

The existing literature on the effect of medical comorbidities and chronic pain conditions on treatment decisions for chronic pain conditions in older adults is sparse (32, 46). The literature suggests that practitioners have a limited understanding of the scale of this problem which is profound. There was no consensus regarding a standard set of comorbidities of relevance. Two studies cited the Charlson comorbidities list which was specifically developed for clinical prognostication in older adults, utilizing this list for the purpose of assessing comorbidities of pain in adults across a broad age-range may not be sufficiently expansive. We show here that there is a small number of studies addressing the co-existence of medical comorbidities and chronic pain conditions and very few examine this phenomenon comprehensively, and we did not identify any other studies that investigate how comorbidities affect pain clinical decision-making. Some studies have asked about comorbidities in other populations, not specifically focusing on older adults—a population where the multiplicity of comorbidities expands the challenge and risk of medication-based management (88, 89). This study offers an important addition in systematically developing a survey instrument designed to characterize the impact of pain comorbidities in older adults on treatment decisions.

### Integrating literature, data, and expert opinion

We utilized a 3-pronged approach to survey instrument development and combined evidence-based methods with the subject-matter expertise of our interprofessional working group, aiming at a robust instrument with clinical and real-world contextual relevance. First, peer-reviewed literature provided the initial framework of comorbidities that was further refined by the professional experience of our advisory group. With their input, the terms *falls*, *dizziness*, and *delirium*, were added due to relevance in the context of our study aims (90–93). *Vitamin D deficiency*, v*itamin B12 deficiency*, and *hepatic impairment* have significant clinical relevance in the treatment of chronic pain-associated conditions, e.g., enthesiopathies, neuropathies, yet were not prominently included in the literature (94–98). When addressing conditions, such as pain, that impact a large percentage of older adults and have profound impacts on many domains of function, it is important to include a diverse range of healthcare professionals in projects which require appraisal and integration of complex data (4, 16). Finally, the utilization of real-world claims data codes provided statistical evidence and confirmation of the prevalence of comorbidities in the United States and further validated inclusion in our instrument (56, 60). A deliberate, interprofessional process led to the development of this research instrument (99).

### Limitations

This is a pilot study describing the use of an intentional interprofessional process to develop a survey to assess pain clinical decision-making in older adults with single highly prevalent comorbidities. Some limitations are noted. The Medicare data which was reviewed by the interprofessional advisory panel was drawn from a demographically representative population of older adults, nonetheless, it is acknowledged that claims data may underestimate or overestimate the prevalence of certain conditions (100–102). Some “conditions” are defined by nonspecific terms, e.g., headache and hypertension, whereas others were more specific such as obstructive sleep apnea and opioid use disorder, so that the broader classes pertaining to these diagnoses, i.e., sleep disturbances and substance use disorders, may not be well captured by the survey (56, 103). This reflects the real world complexity of clinical practice wherein both detailed specification as well as the capacity to abstract to the more general are important skills (104). This data was useful in familiarizing non-medical providers with an estimate of condition prevalence from contemporary data and is intended in this article to provide the reader with actionable data to enhance pain education module development. We did select a “typical” population from the Medicare data focusing on the older adult aged 75–80 who was alive for all of the study year, was enrolled in Part B but not in Medicare Advantage (Part C), and who had between 19 and 30 claims. The latter restriction was included because we and others have noted that diagnostic rates vary widely with claim rates; the number of claims selected for this study included the median 20% of claims, e.g., claim rates ranging from the 40%ile to the 60%ile as our goal was to evaluate the “median” diagnostic rates for the population. It acknowledged that older adults vary tremendously in terms of health and morbidity so that no single number can capture the full flavor, we seek to present a single number that is representative of the typical morbidity burden in the age group studied. It was challenging to develop an effective literature search strategy. Much of the pain literature focuses on “complex pain”, e.g., temporomandibular joint disorder, but does not address “medical complexity” and pain (105). Several meetings with the healthcare informationist were necessary to develop an effective strategy which ultimately included searching for pairs, i.e., a pain condition paired with a medical condition, for several of the high prevalence conditions. Although an effort was made to include several professions in the study group, the study team was led by two specialty physicians whereas the workforce for primary care is increasingly comprised of a broader range of healthcare providers including nurse practitioners, physical therapists, and physician assistants (106–108). This pilot study included a limited number of study subjects and a larger scale test of this instrument is underway, this report serves to explain the construction of the instrument and report feasibility (99). Finally, this study examines the impact of single comorbidities, however it is common for older adults, especially those of advanced age, to experience multiple serious health conditions simultaneously, i.e., multimorbidity, and to face health system challenges in coping with the medical instructions and treatments, i.e., multicomplexity (3, 46). We posit that clinical decision-making burdens likely increase as comorbidities multiply, thus it is important to examine the impact of multimorbidity and multicomplexity, it is our intention that this study provides an important foundation for that future work.

## Conclusions

Comorbidities such as dementia, depression, anxiety, opioid use disorder, dizziness, falls, delirium, congestive heart failure, stroke, hypertension, diabetes, renal and hepatic impairment are likely to have a strong influence on clinical decision-making for healthcare providers working to address pain in older adults. Relatively understudied, the prevalence and impact of comorbidities present in older patients with pain should be proactively incorporated when creating educational curricula; in addition, the impact on clinical guidelines merits substantive consideration. Our survey instrument may be useful to those engaged in pain education research and content development, and improved understanding of pain-related clinical reasoning. We have provided the scoping Medicare data here so that educators can use this information to immediately begin to build more realistic cases incorporating the most common and impactful pain comorbidities. We conclude that further study is essential, and we propose the use of surveys, data analytics, focus groups, and literature reviews as well as systematic development and study of educational materials dedicated to improved clinical pain care, especially focusing on the question of how varying comorbid complexity impacts the decision-making processes of clinicians caring for older adults with pain.

## Data Availability

The data analyzed in this study is subject to the following licenses/restrictions: The dataset is restricted by an existing data use agreement. Requests to access these datasets should be directed to beth.hogans@va.gov.
